# Cutting Through Complexity: Surgical Management of Severe Palmoplantar Keratoderma

**DOI:** 10.7759/cureus.65768

**Published:** 2024-07-30

**Authors:** Muhammad Taimour Khan, Ibrahim Amjad, Muhammad Rahab Khan

**Affiliations:** 1 Internal Medicine, Combined Military Hospital (CMH) Lahore Medical College and Institute of Dentistry, Lahore, PAK; 2 Plastic Surgery, AmjadPlastics, Miami, USA; 3 Dermatology, I.K. Akhunbaev Kyrgyz State Medical Academy, Bishkek, KGZ

**Keywords:** keratosis palmaris et plantaris, diffuse palmoplantar keratoderma, pediatric surgery, plastic surgery, palmoplantar keratoderma, olmsted syndrome

## Abstract

Olmsted syndrome is a rare genetic disorder characterized by severe thickening of the palms and soles, often resistant to conventional treatments. We present the case of a patient with Olmsted syndrome with a 16-year follow-up. The patient presented at five years of age with treatment-resistant palmoplantar keratoderma despite three years of dermatological management, leading to complications. Surgical interventions included initial debridement down to the deep dermis, which resulted in recurrence after three months. This was followed by a decision for extensive excision down to the subcutaneous tissue, use of a bilayer wound matrix dressing followed by negative pressure wound therapy, and a thin split-thickness graft, resulting in full resolution. The patient, now a college student, has regained normal daily activities. This case underscores the challenges and highlights a novel surgical approach for managing Olmsted syndrome, demonstrating a 16-year follow-up and aiming to improve patient outcomes in these complex cases.

## Introduction

Palmoplantar keratoderma (PPK) is characterized by a widespread epidermal proliferation affecting the palms and soles. Also known as ‘keratosis palmaris et plantaris’ or ‘Olmsted syndrome,’ PPK can be classified based on clinical presentation into diffuse, focal, and punctate types. It can also be classified as acquired or congenital [1]. Recent advancements in genetic studies have shed light on the genetic nature of congenital forms, aiding in diagnosis, screening, and management [2,3].

Diffuse PPK does not have a documented prevalence in the USA, reflecting its rarity. As a result, determining an exact prevalence rate is challenging. However, a notable prevalence of 4.4 cases per 100,000 population in Northern Ireland was documented which typically begins in the first few months of life and fully develops by 3-4 years of age [4]. Diffuse hereditary PPKs appear in early childhood with redness on the palms and soles. Over time, these areas thicken and develop a yellowish, waxy look. There is a distinct border between the affected and unaffected skin, often with a red edge. This condition typically becomes noticeable by ages 3 to 4 [1].

Genetically, PPK is associated with mutations in the KRT9 and KRT1 genes, which express keratin 9 and keratin 1 in the suprabasal palmoplantar skin. The most common mutation involves keratin 9, which also forms heterodimers with keratin 1 to create intermediate filaments in the skin. Subtypes of diffuse PPK can be inherited in autosomal dominant, autosomal recessive, or both patterns [2].

Treatment options for PPK are divided into medical and surgical categories. Currently, surgical options are limited and have varying degrees of success; therefore, medical treatment is the standard of care in most cases. Medical treatments include emollients, topical keratolytic agents (e.g., 6% salicylic acid or urea-based), topical retinoids, topical vitamin D ointment, or oral retinoids [1]. Surgical first-line treatment with debridement provides temporary relief. Secondary infections, common in complex cases, require appropriate treatment with antibiotics and, in some cases, antifungal therapy.

The surgical management for PPK is limited and to the best of our knowledge, the unique method employed in this case has never been used. In one case, skin grafts were employed for treating PPK, primarily using the cross-leg graft method. This approach was utilized either for necrotic areas where a simple skin graft was insufficient or as the primary surgical method, with skin sourced from the calf or thigh. All reported cases had successful outcomes, though the duration of post-treatment success was not specified [5]. In another case, a pediatric patient was treated with a split-thickness skin graft instead of a cross-leg graft due to the patient's young age. The treatment was reported as successful but again showed less data about the follow-up [6]. In another case involving a woman, a split-thickness skin graft was performed specifically on her hands, the affected area. The procedure was successful, and six months later, she was able to resume her normal daily activities [7].

## Case presentation

**Figure 1:**
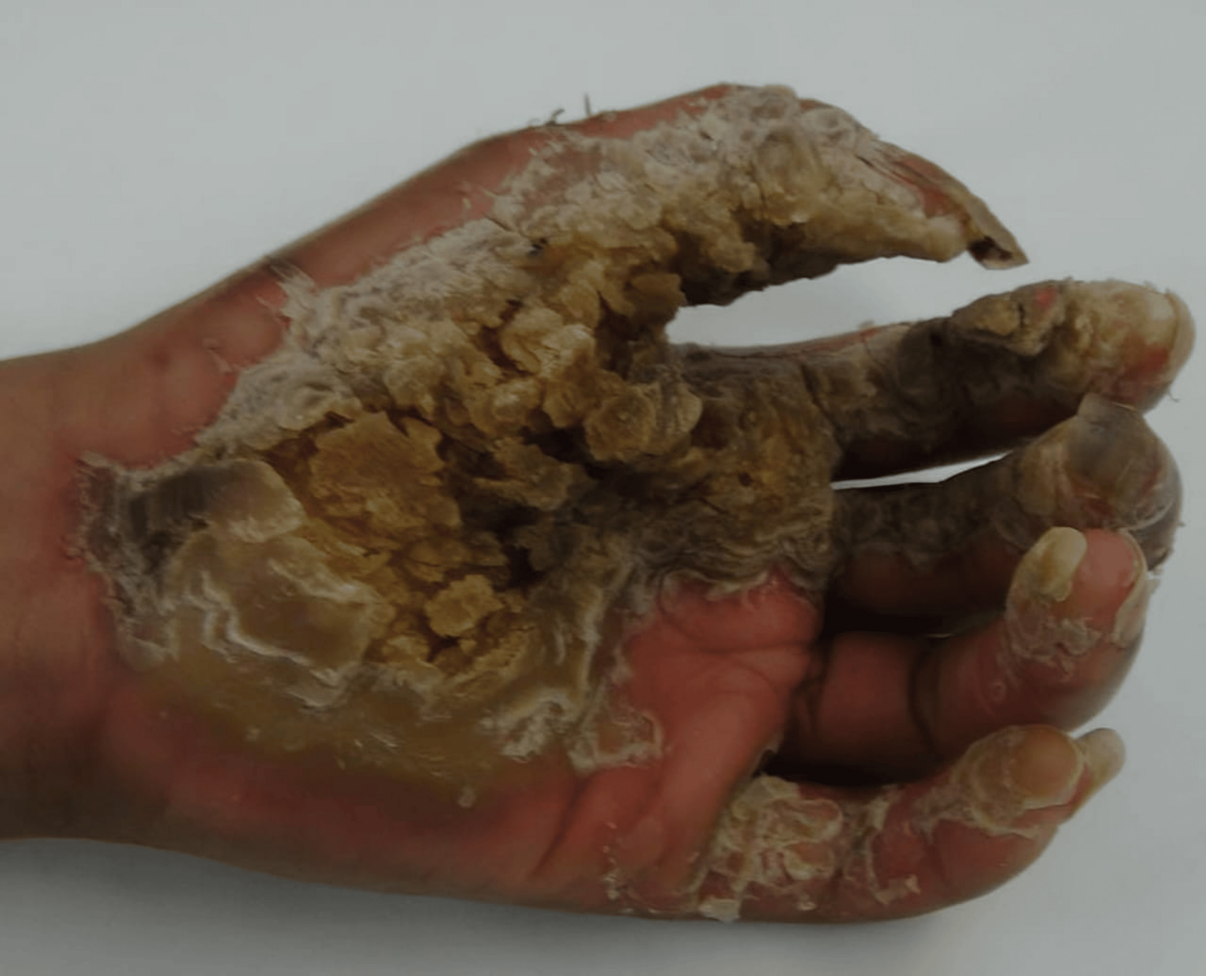
Palmoplantar keratoderma of the hand.

**Figure 2:**
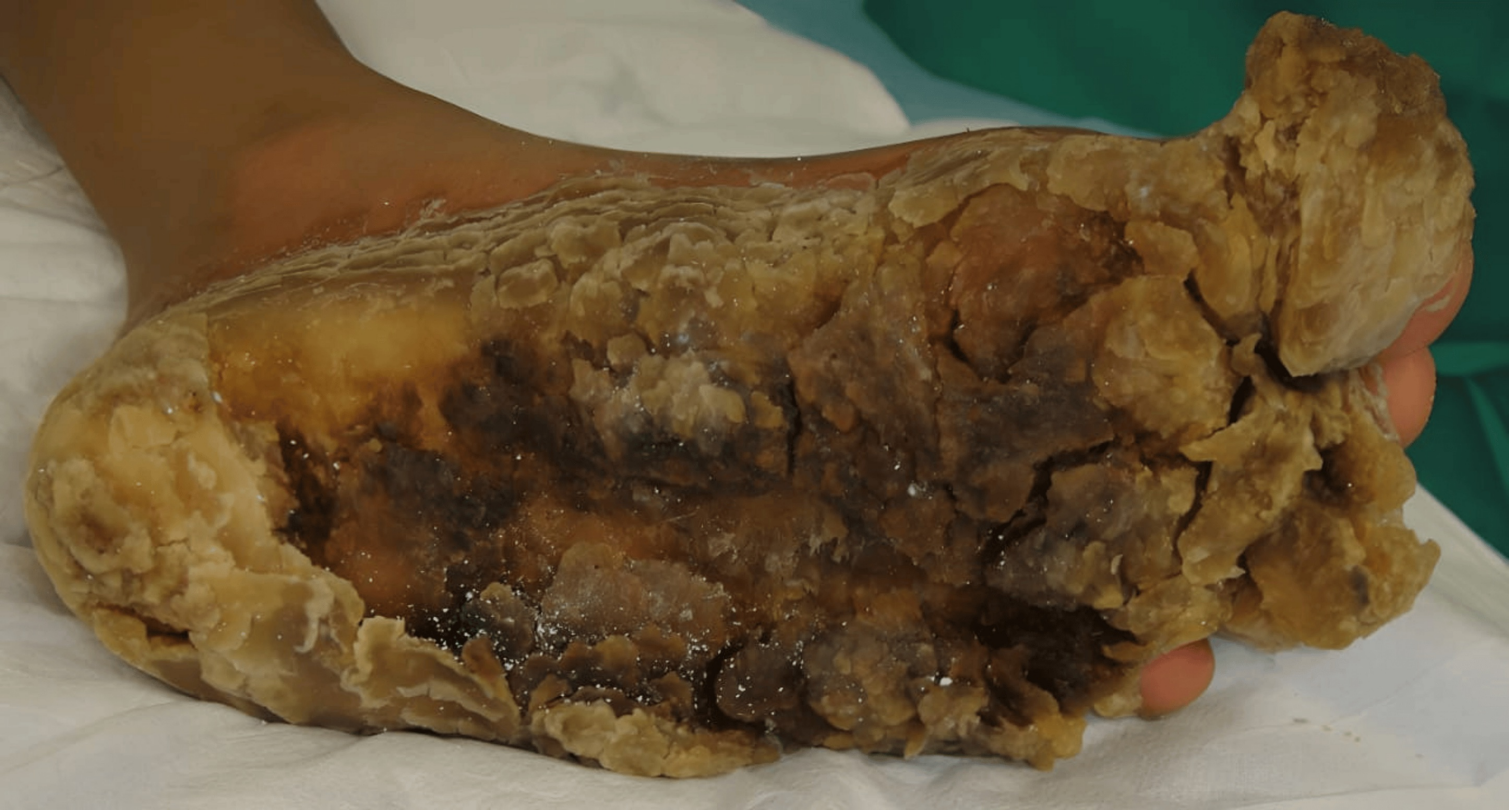
Palmoplantar keratoderma of the patient's foot.

The patient presented as a five-year-old female with no prior medical problems except those related to her PPK as can be seen in Figure 1 and Figure 2. The patient had been actively seen by Dermatology Department, who were attempting to decrease her PPK to the point where it was manageable. Three years prior to getting surgical management, the patient had been admitted multiple times due to severe infection with systemic complications and sepsis. Her feet were also painful and were treated for intractable pain. The patient was confined to a wheelchair. The patient had tried multiple topical treatments and soaks with no improvement. Areas of cracking, pain, and infection were present on her first visit. 

**Figure 3:**
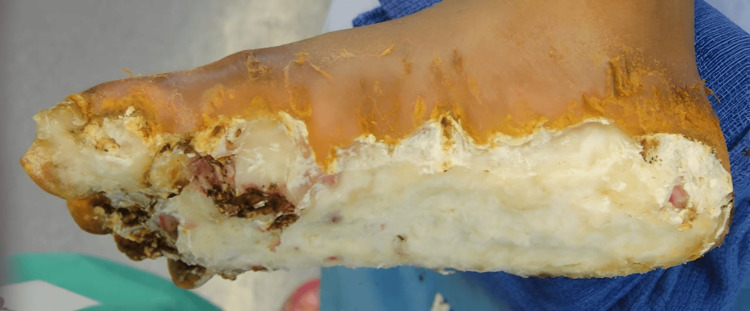
Patient's foot after a surgical debridement using a harmonic scalpel.

**Figure 4:**
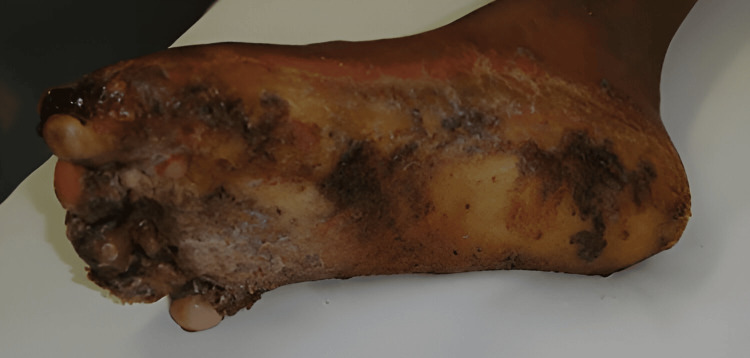
Recurrence 3-4 months later, after the last surgical debridement.

**Figure 5:**
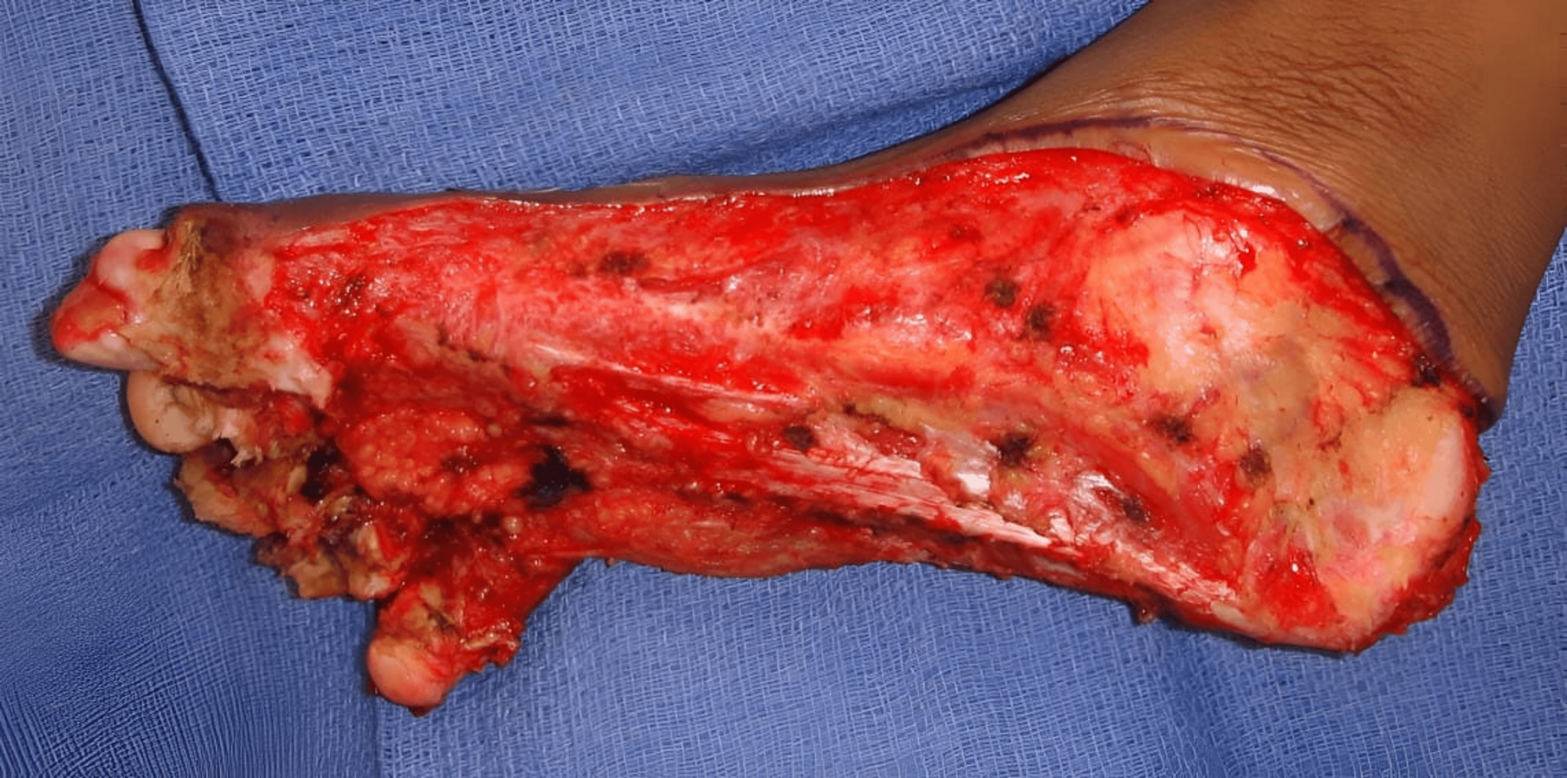
Excision of tissue down to the subcutaneous layer.

**Figure 6:**
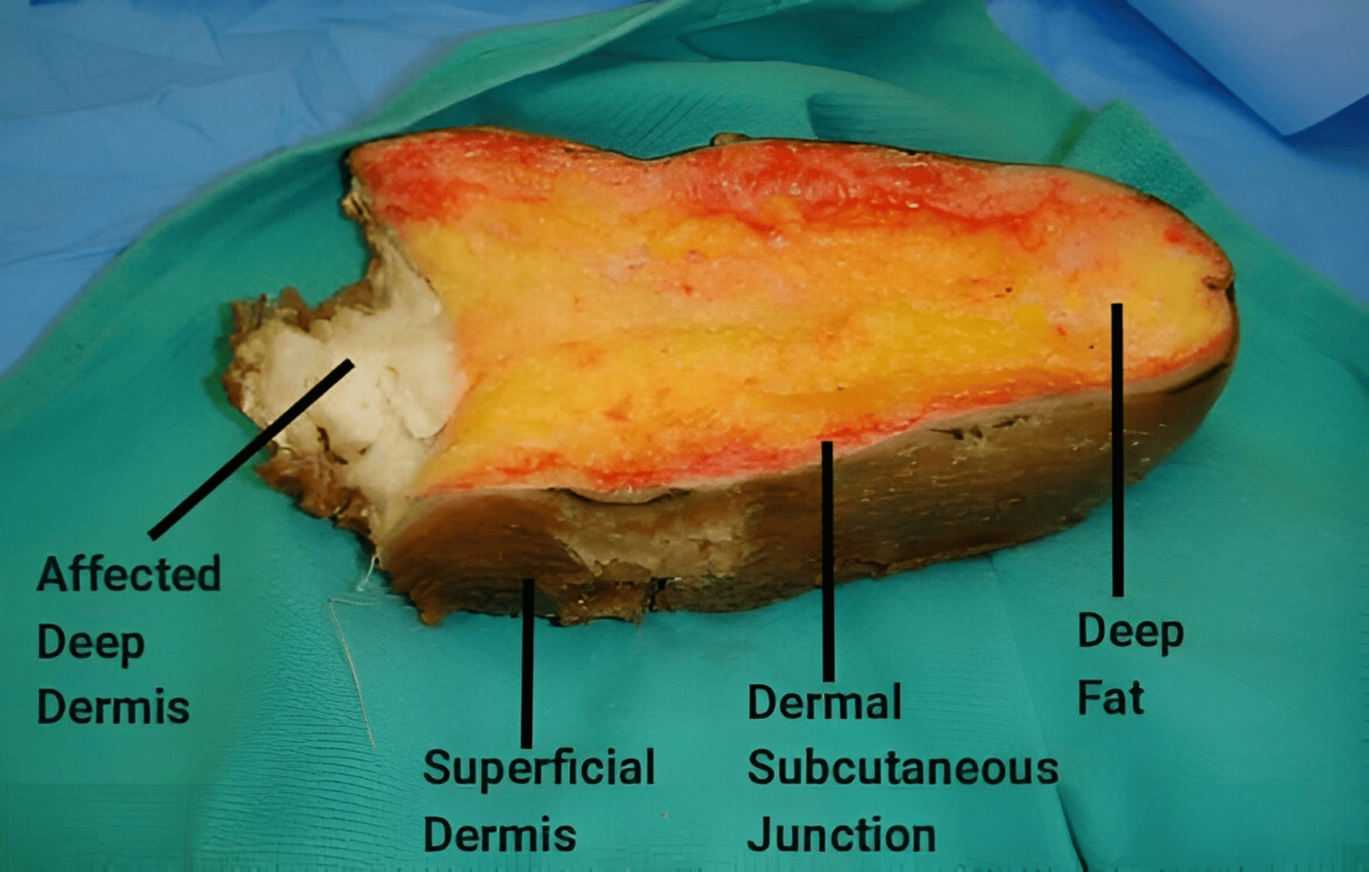
Labeled resection specimen.

**Figure 7:**
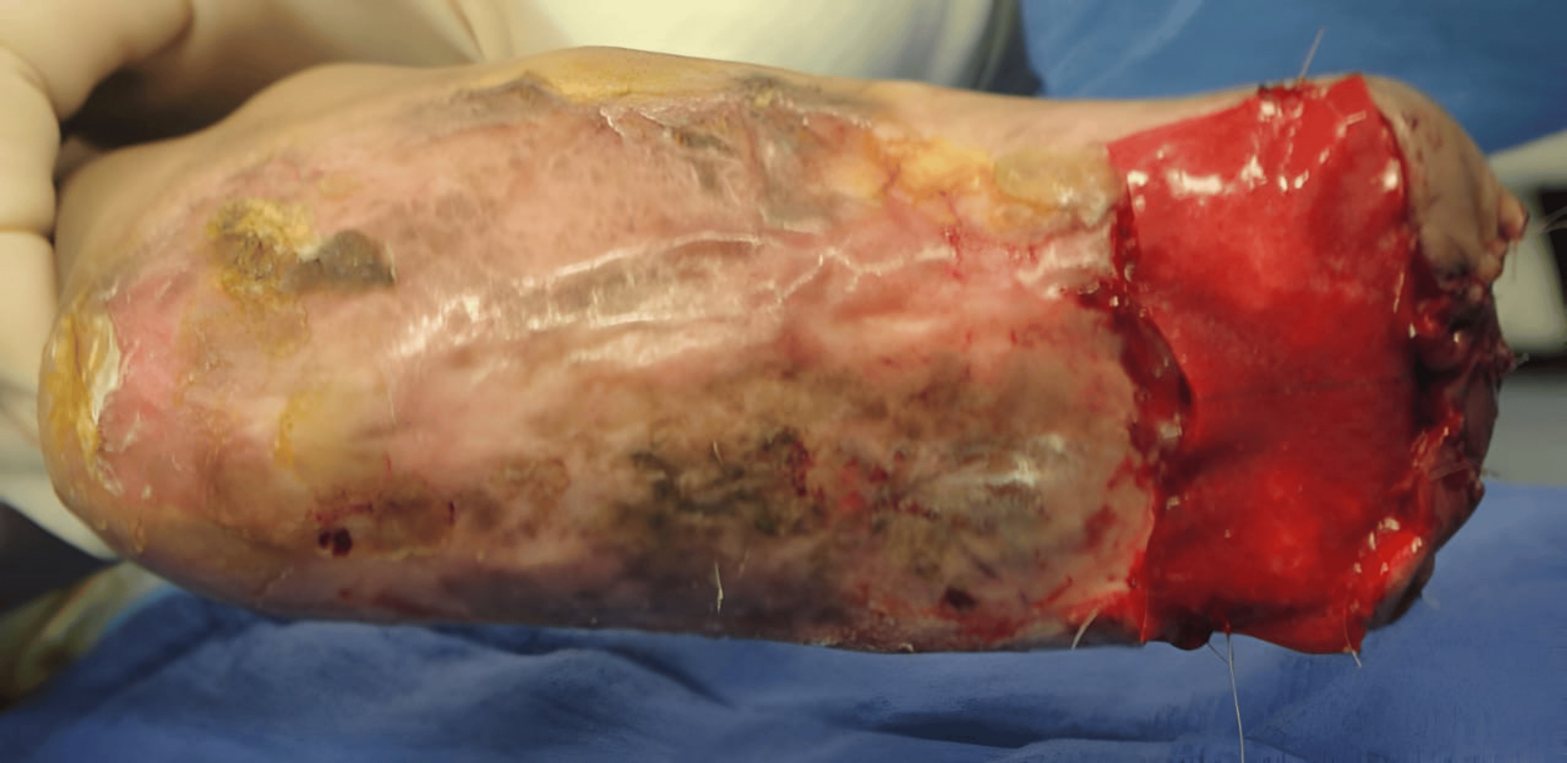
Foot after placing a double layer of integra and then covered by split-thickness graft.

Debridement was initially discussed. The patient had some debridement done sharply, but the keratoderma subsequently thickened rapidly. Deeper debridement was presented as an option. With the use of the harmonic scalpel and a smoke evacuator debridement was carried out into the deep portions of the dermis as can be seen in Figure 3. Initially, this was an effective procedure, allowing the patient to remain without thickened changes, infection, smell, or pain for approximately 3 to 4 months. Several of these debridement procedures were done. Approximately 3 to 4 months after the last debridement, recurrence was rapid as can be seen in Figure 4. The patient who had been able to walk was again wheelchair bound. The decision was made to excise the keratoderma en bloc, removing the dermis and subcutaneous tissue, as shown in Figure 5. The excised tissue can be seen in Figure 6, which reveals the plantar fascia of the feet. After excision, Integra would be placed with negative pressure wound therapy (NPWT), followed by a second layer of Integra with NPWT, allowing for a more durable sole of the foot. Final coverage would be achieved with a thin split-thickness graft, as shown in Figure 7. The risks and complications of this aggressive treatment were understood, and one foot was operated on first. The surgery went successfully, and the patient was able to bear weight after the skin graft fully healed.

**Figure 8:**
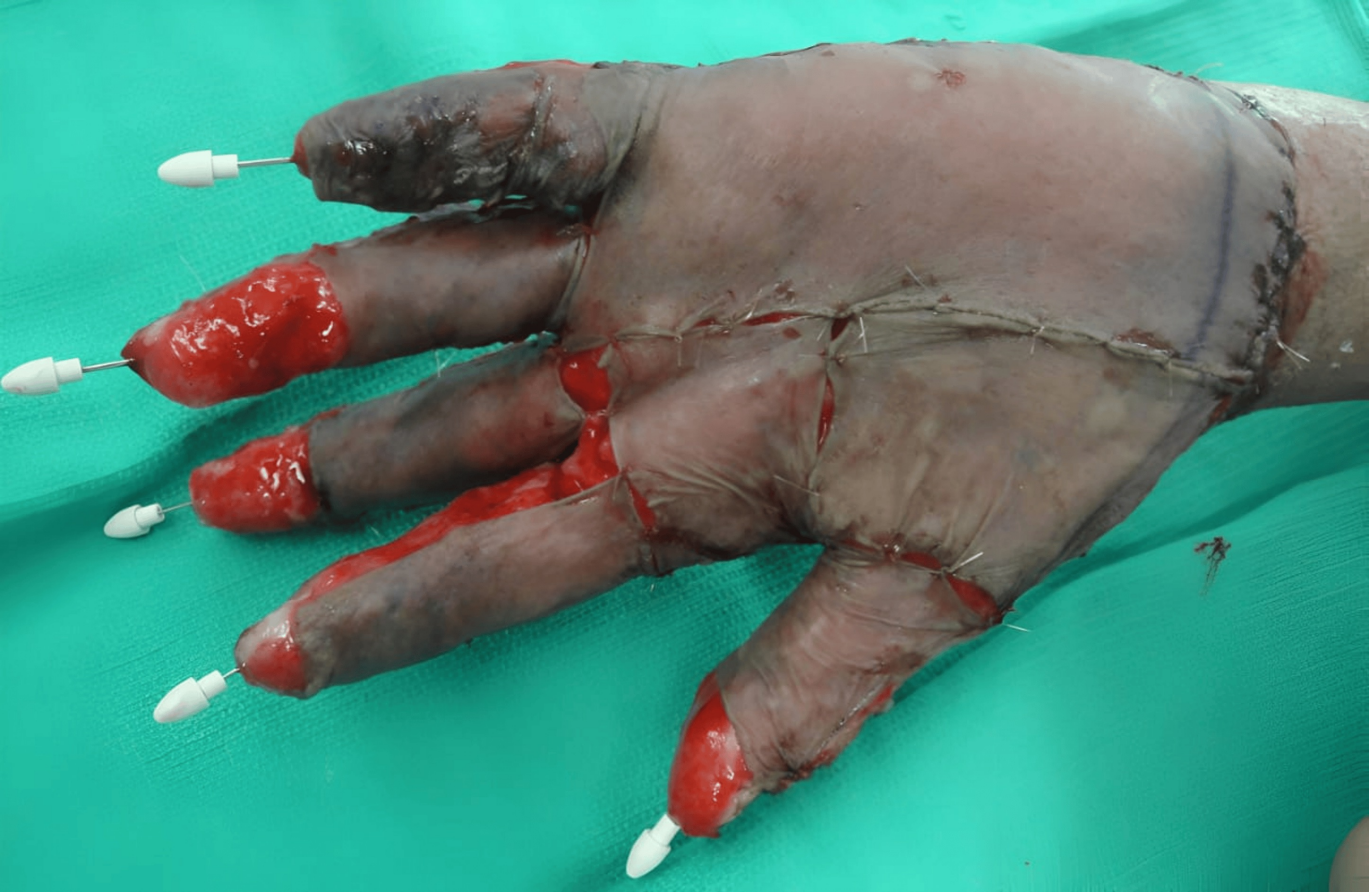
Patient's hand covered by split-thickness graft.

Attention was then turned to the other foot, which was also successfully treated in the same fashion. With success noted in the feet, attention was turned to the hands, starting with the right hand. The procedure was done in a similar fashion, down to the palmar fascia, and extending to the subcutaneous tissue of the fingers. Great care was taken not to damage digital arteries or nerves. The patient’s fingers were pinned straight to decrease the possibility of contracture. After NPWT and granulation, a thin split-thickness skin graft was placed as can be seen in Figure 8. This procedure was repeated for the contralateral side. 

**Figure 9:**
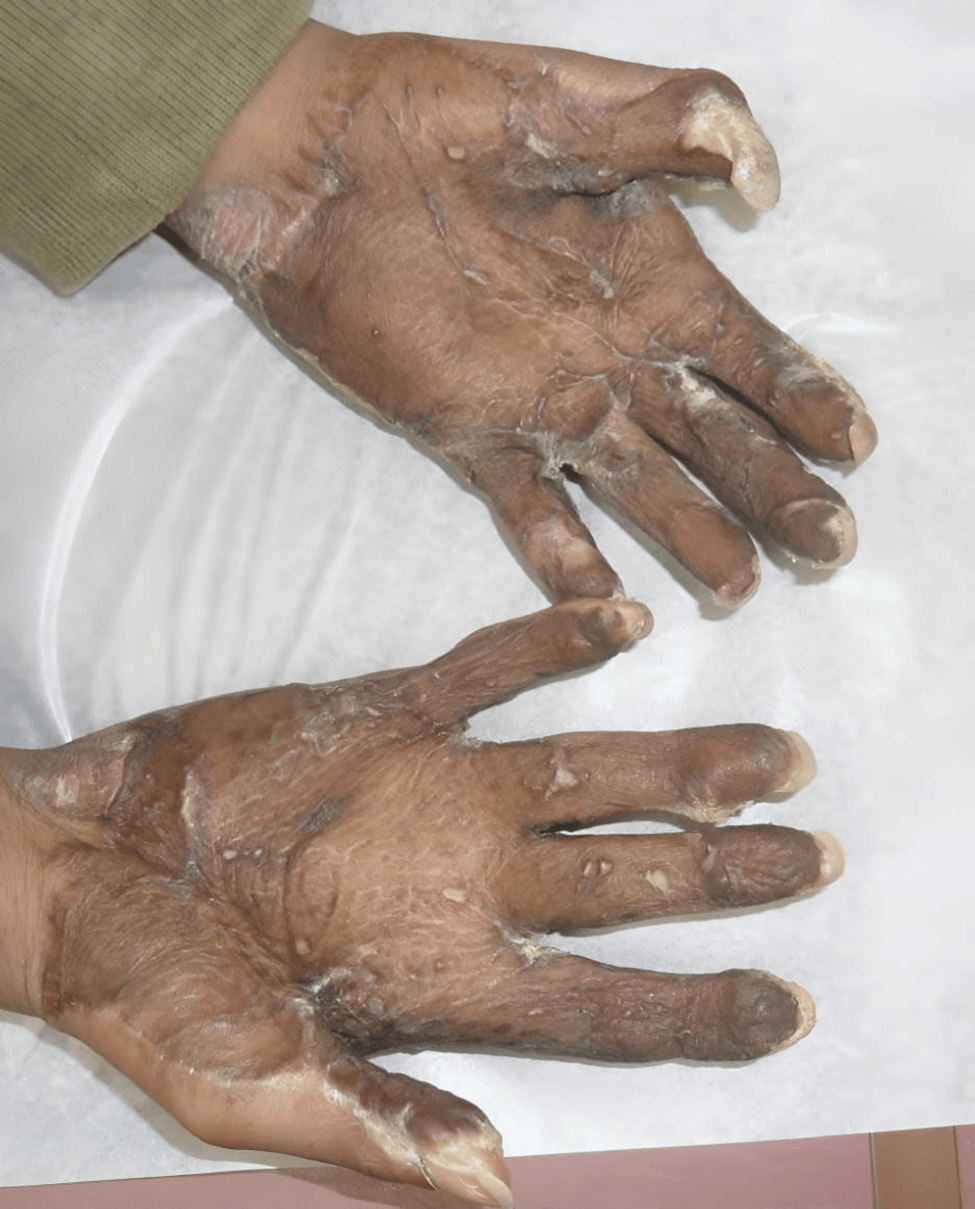
16-year follow-up of the patient's hands.

**Figure 10:**
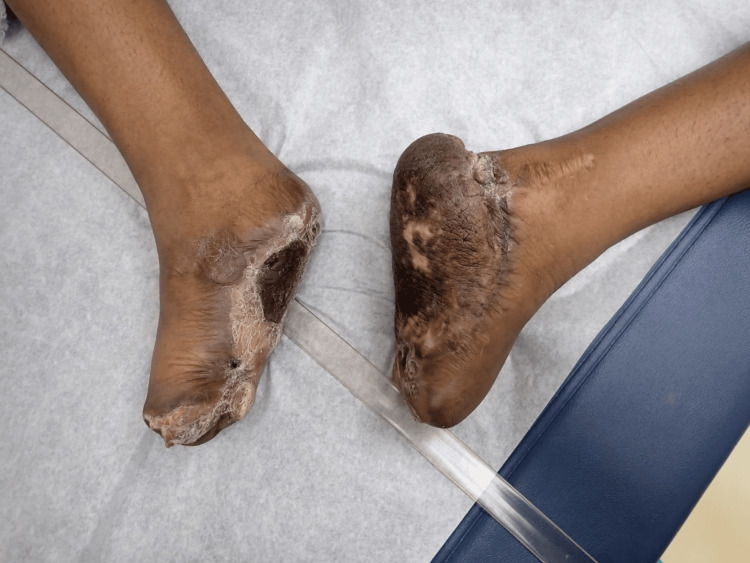
16-year follow-up of the patient's feet.

The patient was monitored carefully. Some areas of minimal recurrence were noted, less than 1 cm (about 0.39 in) in diameter and were treated with wound care with topical urea. When one area did progress, a small resection was done followed by skin grafting. After six months of walking as a middle school student, there was breakdown of the heel to the plantar fascia. Concern for osteomyelitis and progression of the wound prompted the use of a rectus free flap to allow for significant padding in this portion of the heel followed by a conventional split-thickness skin graft. The flap was monitored appropriately and shrank with no recurrence. After 16 years, the patient now walks and is currently a college student with the stability of her hands as can be seen in Figure 9 and feet as can be seen in Figure 10. She is able to take notes and type, with an almost full range of motion of her fingers. The patient states that she is able to lead a normal and productive life.

## Discussion

Here, we presented the case of a female patient who was unsuccessfully managed with conventional treatments for PPK, later developing severe complications including pain, infection, and sepsis. Initially, debridement was done using a harmonic scalpel down to the deep dermis which showed temporary improvement. The harmonic scalpel which uses ultrasonic vibrations causes less thermal damage to surrounding tissues and helps to reduce bleeding with a smoke evacuator removing the smoke from the cauterized tissue. Later, the condition not only recurred but also worsened. Therefore, a nuanced and careful approach was adopted by excising down to the level of the plantar fascia, removing both the dermis and subcutaneous tissue. To the best of our knowledge, such an approach has not been previously implemented. Other reported surgeries typically involved excision including the dermis and often failed to provide long-term follow-up data confirming the absence of recurrence [5,6]. Recent studies suggested a genetic basis for PPK [2], indicating that the problem may arise from the suprabasal region of palmoplantar skin, these studies were more focused on diagnosis and have a promising future in genetically centered treatment approaches, such as using RNA interference (RNAi) [3], rather than evaluating surgical outcomes.

Genetic studies have a promising impact on understanding the nature of PPK, as well as its treatment, diagnosis, and prevention of complications. Through KRT-9 and KRT-1, we can understand the pattern of diffuse-type PPK [2]. RNA interfering (RNAi) has been successfully performed on mice, showing positive outcomes [3]. The development of esophageal carcinoma due to palmoplantar keratosis is associated with a genetic mutation (RHBDF2 on 17q25.1) with the autosomal dominant pattern. Screening is recommended in the late fifties, with annual follow-ups thereafter [8]. One study performed plastic surgery on an adult patient using the cross-leg flap method and reported success but had absent follow-up data [5]. The cross-leg flap has utility in the feet with padded tissue with the skin but comes with significant donor site morbidity. Also, a cross-leg flap is not able to be used for the treatment of the hands. Another study involving a pediatric patient used a split-skin graft instead, as using a cross-leg graft on a child was deemed too difficult, with limited long-term follow-up data. They monitored the patient from April until discharge in October, treating with debridement, and then with recurrence, they did plastic surgery [6]. A similar concern was held during a careful discussion of the surgical approach of this case, as the cross-leg graft would require the child to undergo more operations and endure greater discomfort. This method necessitates prolonged periods of donor and recipient site proximity and extended immobilization. 

Medical therapies are the gold standard in non-severe cases. As has been noted in this case, progression commonly occurs requiring a more aggressive approach. Surgical scenarios have shown promising results. The novel approach in this case was to perform a full excision including the subcutaneous level, unlike other cases, excision was done down to the level of the dermis [5-7]. Due to the severity and possibility of recurrence, most studies that excised the dermis failed to report post-operative success timelines. Only one study reported post-operative success with no recurrence after six months, but the pathology of PPK was limited to her palms only [7]. 

Using a stepwise progression with family-centered care, along with input from the patient and family, was paramount to the treatment of this condition. Given the severity of this case, conventional treatments were exhausted. Due to the patient’s wheelchair-bound condition and her inability to complete activities of daily living, a more extreme intervention was required. The use of a bilayer dermal matrix and NPWT in two layers allowed for a more durable contact surface. This dermal substitute also facilitated the use of thin split-thickness skin grafts. Thin split-thickness skin grafts allow for re-harvesting of the donor site, which is necessary in a case like this where multiple grafts are required.

Given the patient’s limited ability to walk, one of her feet could not sustain the trauma of walking, necessitating the introduction of new tissue in the form of a free flap. Moreover, this case extended further than other studies with a full 16-year postoperative follow-up. The young patient is now a successful college student with a good range of motion and no recurrence of issues in either her hands or feet. She is able to lead a normal life, performing her activities of daily living and more.

## Conclusions

In this case report, we detail the successful management of a patient with severe PPK, who experienced complications including pain, infection, and sepsis following conventional treatments. Our innovative surgical approach involved excising tissue down to the plantar fascia, beyond the dermis and subcutaneous layers, a method not previously documented. This approach contrasts with other surgeries which typically only excised the dermis and lacked long-term follow-up data. Moreover, most of the studies failed to do an adequate follow-up that showed full resolution with no recurrence and in our case, a 16-year follow-up was successfully done. For severe cases, our focus on an extensive surgical intervention proved effective. The patient has shown a return to normalcy and no recurrence, underscoring the potential of comprehensive surgical management for this condition.
